# Maximum cardiac performance of Antarctic fishes that lack haemoglobin and myoglobin: exploring the effect of warming on nature’s natural knockouts

**DOI:** 10.1093/conphys/coz049

**Published:** 2019-10-11

**Authors:** Stuart Egginton, Michael Axelsson, Elizabeth L Crockett, Kristin M O’Brien, Anthony P Farrell

**Affiliations:** 1 School of Biomedical Sciences, University of Leeds, Leeds LS2 9JT, UK; 2 Department of Biological and Environmental Sciences, University of Gothenburg, Gothenburg SE-405 30, Sweden; 3 Department of Biological Sciences, Ohio University, Athens, OH OH45701, USA; 4 Institute of Arctic Biology, University of Alaska Fairbanks, Fairbanks, AK AK99775, USA; 5 Department of Zoology, University of British Columbia, Vancouver, BC V6T 124, Canada

**Keywords:** Cardiac output, cardiac work, heart rate, icefishes, thermal adaptation, warming

## Abstract

Antarctic notothenioids, some of which lack myoglobin (Mb) and/or haemoglobin (Hb), are considered extremely stenothermal, which raises conservation concerns since Polar regions are warming at unprecedented rates. Without reliable estimates of maximum cardiac output ($\dot{Q}$), it is impossible to assess their physiological scope in response to warming seas. Therefore, we compared cardiac performance of two icefish species, *Chionodraco rastrospinosus* (Hb^−^Mb^+^) and *Chaenocephalus aceratus* (Hb^−^Mb^−^), with a related notothenioid, *Notothenia coriiceps* (Hb^+^Mb^+^) using an *in situ* perfused heart preparation. The maximum $\dot{Q}$, heart rate (*f*_H_), maximum cardiac work (*W*_C_) and relative ventricular mass of *N. coriiceps* at 1°C were comparable to temperate-water teleosts, and acute warming to 4°C increased *f*_H_ and *W*_C_, as expected. In contrast, icefish hearts accommodated a higher maximum stroke volume (*V*_S_) and maximum $\dot{Q}$ at 1°C, but their unusually large hearts had a lower *f*_H_ and maximum afterload tolerance than *N. coriiceps* at 1°C. Furthermore, maximum *V*_S_, maximum $\dot{Q}$ and *f*_H_ were all significantly higher for the Hb^−^Mb^+^ condition compared with the Hb^−^Mb^−^ condition, a potential selective advantage when coping with environmental warming. Like *N. coriiceps*, both icefish species increased *f*_H_ at 4°C. Acutely warming *C. aceratus* increased maximum $\dot{Q}$, while *C. rastrospinosus* (like *N. coriiceps*) held at 4°C for 1 week maintained maximum $\dot{Q}$ when tested at 4°C. These experiments involving short-term warming should be followed up with long-term acclimation studies, since the maximum cardiac performance of these three Antarctic species studied seem to be tolerant of temperatures in excess of predictions associated with global warming.

## Introduction

A remarkable trait among vertebrates is the evolutionary loss of haemoglobin (Hb) and myoglobin (Mb) expression within the Channichthyid family of Antarctic notothenioid fishes (Holeton 1970). Mutations leading to the Hb^−^ state occurred once during evolution of icefishes, ~8.5 MYA (Near, 2004), subsequent to cooling of the Southern Ocean. Loss of Mb expression occurred four times during radiation of the Channichthyids (Sidell *et al*., 1997), resulting in 6 of the 16 species of icefishes lacking Mb in their heart (Sidell *et al*., 1997). Thus, the Hb^−^Mb^+^ and Hb^−^Mb^−^ states are unique to icefishes (Sidell and O'Brien, 2006), although loss of Mb expression is a trait shared by a few temperate teleosts (Grove & Sidell, 2002; Macqueen *et al*., 2014) whose hearts can function adequately provided blood PO_2_ is sufficient (Bailey *et al*., 1990).

The Hb^−^ and Mb^−^ condition is thought to have restricted Channichthyid distribution primarily to the thermally stable offshore waters of Antarctica, which hover near −1.8°C below the surface year-round. While cold water may be key to survival of stenothermal species by ensuring high levels of dissolved oxygen, and presumably low oxygen requirements, the Western Antarctic Peninsula region is warming at an unprecedented rate. Thus, endemic species possessing modest thermal plasticity (e.g. Bilyk *et al*., 2018) may be especially vulnerable to short-term extreme temperature fluctuations, as well as long-term climate warming (Clarke *et al*., 2007; Beers *et al*., 2010). Given the limited options for altered geographic distribution, documenting scope for performance provides critical insight into capacity to accommodate shifts in environmental temperature, and hence the potential for longer-term adaptations necessary to allow stock conservation within the current ecological niche.

In terms of physiological performance, cardiac performance is viewed by some as playing a critical role in setting the upper thermal limits of temperate-zone fishes (Wang and Overgaard, 2007; Portner and Farrell, 2008; Farrell, 2009, 2016; Eliason *et al*., 2011), though others have challenged this contention (Ern *et al*., 2016). Indeed, extensive adaptational remodelling of the icefish cardiovascular system is apparent for life in the frigid, but stable Southern Ocean. For example, the 90% decrease in oxygen-carrying capacity associated with the Hb-free blood of icefishes (Ruud, 1954) has been compensated for by a thin-walled ventricle with relative mass 10-times larger than other benthic teleosts (Tota *et al*., 1991; Farrell and Jones, 1992; Axelsson, 2005; Farrell and Smith, 2017) that can eject a very large stroke volume (*V*_S_) and produce an exceptionally high cardiac output ($\dot{Q}$) for an animal living at ~ 0°C (Hemmingsen and Douglas, 1977). The concern, however, is that a very large routine $\dot{Q}$ needed to offset a low blood oxygen-carrying capacity might leave little cardiac scope for activity or facing environmental challenges such as warming.

Earlier studies with icefishes have reported on the maximum $\dot{Q}$ (an integrative measure limiting fish activity), but its response to warming is poorly documented. For example, maximum $\dot{Q}$ in *Chionodraco hamatus* (Hb^−^Mb^+^) is impressive at 3°C: ~ 300 ml min^−1^ kg^−1^ (Tota *et al*., 1991; Acierno *et al*., 1997). This maximum performance index is best measured with a working perfused heart given the difficulty of inducing a maximum performance of icefish *in vivo* (see Joyce *et al*., 2018b). To determine cardiac scope also requires reliable routine $\dot{Q}$ values, but these vary considerably, as pointed out by Joyce *et al*., (2018b), who also reported routine $\dot{Q}$ for *Chaenocephalus aceratus* (Hb^−^Mb^−^) at 0.8°C that was lower than previously reported and increased with both moderate activity and with acute warming. By comparison, routine $\dot{Q}$ is just 6 ml min^−1^ kg^−1^ at ~ 0°C in *N. coriiceps* (Hb^+^Mb^+^), which has an haematocrit of ~ 16% (Egginton, 1997; Joyce *et al*., 2018b), although other red-blooded notothenioids of different ecotype and size have a higher routine $\dot{Q}$ (Axelsson, 2005). Moreover, *N. coriiceps* can increase routine oxygen uptake, $\dot{Q}$, *V*_S_ and *f*_H_ appreciably after warm-acclimation (Egginton and Campbell, 2016; Joyce *et al*., 2018a). Other cardiovascular adaptations of icefishes include wide-bore arteries and capillaries that reduce overall systemic vascular resistance to just 10–20% that of Hb^+^ fishes, and lower arterial blood pressure (Egginton, 1997) and cardiac afterload (Egginton *et al*., 2002). Thus, mass-specific cardiac work (*W*_C_) in icefishes is estimated to range between 0.35 and 2.3 mW g^−1^ (Axelsson, 2005), at the lower end of the range for teleost fishes (Farrell and Smith, 2017).

Therefore, indications exist that icefishes may have a reasonable cardiac scope and a moderate capacity to respond to warming, but without robust estimates of maximum $\dot{Q}$ for an Hb^−^Mb^−^ icefish we cannot test these possibilities. Indeed, maximum cardiac performance has never been assessed in *C. aceratus*. Therefore, to better understand how loss of Mb and Hb expression might affect vulnerability to ocean warming, we hypothesized that warming impairs maximum cardiac performance of icefishes, especially when compared with the heart of a species possessing Hb and Mb. To test this hypothesis, we compared maximum cardiac performance at 1°C and 4°C for three Antarctic notothenioids that naturally express different levels of Hb and Mb. We reasoned that the loss of facilitated oxygen transport, either by systemic convection (Hb) or cellular diffusion (Mb), would limit maximum cardiac capacity. In comparing two icefish species that are Hb^−^ the influence of Mb would be revealed, and the influence of Hb evident by comparison with a red-blooded nototheniid.

## Material and methods

### Collection of animals

Adult *Chaenocephalus aceratus* (Hb^−^Mb^−^), *Chionodraco rastrospinosus* (Hb^−^Mb^+^) and *Notothenia coriiceps* (Hb^+^Mb^+^) were collected around the Western Antarctic Peninsula during the austral fall and winter of 2015 (see O’Brien *et al*., 2018). They were held at Palmer Station in ambient seawater temperature (0 ± 1°C) for a maximum of 3 weeks before experimentation. *N. coriiceps* were fed cubes of fish muscle every other day, but not 24–48 h prior to surgery. The icefishes did not feed in captivity; they are known to maintain a lower metabolic rate than other notothenioids and likely grow sporadically, feeding at relatively broad but seasonally dependent time intervals (Johnston and Battram, 1993; Campbell *et al*., 2008).

### The *in situ* heart preparation

We adopted a well-established *in situ* working, perfused heart preparation that has been used across a wide variety of fish species, including Arctic fish at low temperatures (e.g. Farrell *et al*., 1988, 2013). This preparation retains full anatomical integrity of the heart and generates a maximum $\dot{Q}$ equivalent to that observed *in vivo*. Output pressure can be raised to determine the maximum pressure-generating ability and maximum *W*_C_ (Farrell *et al*., 1991). All experiments were approved by the University of Alaska, Fairbanks Institutional Animal Use and Care Committee (570217-18).

Briefly, anaesthetized fish (Egginton and Campbell, 2016) were maintained on a surgical operating sling by continuously irrigating the gills with ice-chilled seawater containing MS-222 (50 mg l^−1^). Heparin (~100 i.u. kg^−1^ in saline) was injected into the caudal vessels to prevent blood clots. A mid-line ventral incision, occlusion of the gut arterial blood supply with umbilical tape and removal of the intestines provided access to the hepatic veins, all but one of which were ligated. A saline-filled input cannula [connected to a Marriot bottle containing physiological perfusate (see below) bubbled with 100% oxygen] was advanced into the sinus venosus *via* the remaining vein and secured in place. Air bubbles sucked into the heart when this vein was cut were prevented from entering gill vessels (which would restrict $\dot{Q}$) by severing the ventral aorta at the isthmus and anterior to the pericardium prior to the cut. The Marriot bottle height was adjusted so that $\dot{Q}$ was not excessive and the exceptionally thin-walled cardiac chambers were not over filled. Next, the output cannula was inserted into the severed ventral aorta and secured. The ducts of Cuvier were then occluded so that the heart only received perfusate; this procedure also crushed all nerves supplying the heart. Within ~ 20 min the heart preparation was transferred to and fully immersed in a saline bath (250 mM NaCl) housed inside a 4°C environmental room. The input cannula was then connected to a pressure head that delivered oxygenated perfusate at a precise cardiac filling pressure (P_i_). The bath and the reservoirs containing oxygenated perfusate were maintained at a constant temperature (either 1°C or 4°C) with a Neslab RTE recirculating water bath (Thermofisher Scientific, Waltham, MA, USA).

Initial $\dot{Q}$ was nominally set at ~ 13 ml min^−1^ kg^−1^ body mass by adjusting P_i_ (typically < 1 cm H_2_O and sometimes sub-ambient; 1 cm H_2_O = 0.1 kPa) to set *V*_S_ while the heart beat with a myogenic rhythm. A constant pressure head device connected to the output cannula set the initial cardiac afterload (P_o_) at a nominal ~ 40 cm H_2_O for *N. coriiceps* and ~ 20 cm H_2_O for both icefish species. Mean dorsal aortic blood pressure is higher for *N. coriiceps* (18.7–42.8 cm H_2_O) than *C. aceratus* (12.3–23.4 cm H_2_O) (Egginton, 1997), but information on *in vivo* ventral aortic blood pressure to set their initial P_o_ is lacking. Regardless, maximum P_o_ was directly measured during the protocol by incremental increases until maximum $\dot{Q}$ began to fail, suggesting this was a reasonable approach, especially since we repeated this response several times for each preparation. These stable perfusion conditions were maintained for at least 10 min prior to assessing maximum cardiac performance. The pericardium was opened at the end of the experiment to ensure proper placement of input and output cannulae and weigh the heart.

### Assessing maximum cardiac performance *in situ*

Protocols to generate maximum *V*_S_, maximum $\dot{Q}$, maximum P_o_ and a maximum cardiac work (*W*_C_) are detailed elsewhere (e.g. Farrell *et al*., 1988). Briefly, incremental increases in P_i_ with a myogenic heartbeat yielded a maximum *V*_S_ and a maximum *Q*. All preparations gave a robust Starling response, similar to other teleost working heart preparations, with *V*_S_ being most sensitive to a P_i_ < 3 cm H_2_O; smaller incremental changes in $\dot{Q}$ occurred up to 5 cm H_2_O and occasionally up to 8 cm H_2_O. (Note: maximum *V*_S_ could be higher *in vivo* if cardiac vagal tonus lowered *f*_H_, but maximum $\dot{Q}$ would not necessarily be greater.) Maximum P_o_ was determined at maximum $\dot{Q}$ by raising P_o_ in 5–10 cm H_2_O increments until maximum *W*_C_ was attained, at which point any increase in P_o_ was matched by an equivalent decrease in $\dot{Q}$. While hearts can generate a higher P_o_, but not without disproportionately decreasing $\dot{Q}$ and *W*_C_, icefishes displayed an interesting phenomenon when P_o_ was increased beyond the maximum *W*_C,_ wherein *f*_H_ abruptly halved, *V*_S_ doubled and maximum $\dot{Q}$ hardly changed; this situation was immediately reversed by reducing P_o_. After maximum cardiac performance had been assessed, routine $\dot{Q}$ and P_o_ were rapidly restored, which quickly re-established a stable routine $\dot{Q}$.

Adrenaline can enhance maximum cardiac performance of perfused teleost hearts (Farrell *et al*., 1986), including Arctic fish at 1°C (Farrell *et al*., 2013). However, low circulating levels of catecholamines in Antarctic fishes, even after moderate stress that rose substantially only *in extremis* (Whiteley and Egginton, 1999), raised the possibility that no adrenergic stimulation would be needed in the present study. Nevertheless, preliminary experiments showed that *C. aceratus* preparations quickly lost pumping capacity unless they had been stabilized with 50 nM adrenaline in the perfusate, which is a low concentration when compared to the levels induced by stress (~5 μM). Accordingly, our initial perfusion conditions, including during surgery, were standardized to include 50 nM adrenaline. Thus, the initial *f*_H_ reported below reflects a tonic adrenergic effect on the myogenic heartbeat. Since the preliminary experiments with all three species showed that adrenaline concentrations > 5 μM elicited no further stimulation of cardiac performance, maximum cardiac performance was assessed with three levels of catecholamine (50nM, 0.5 μM and 5 μM) to provide insight into the modulatory effects of adrenergic stimulation. The need to test a range of concentrations to establish the effect of different levels of adrenergic stimulation reflects a lack of prior data in the literature on these species, so was an essential prerequisite for a robust experimental design. Up to five concentrations have been tested previously without performance deterioration.

### Temperature treatments

The three species were assessed at 1°C, which was near their habitat temperature (0 ± 1°C). Attempts to more closely match test and exposure temperature in a 4°C environmental chamber produced unwanted ice formation. Acute warming to 4°C of heart preparations from fish acclimated to 0 ± 1°C were assessed with the experimental bath and perfusate reservoirs maintained at 4°C), but only with *C. aceratus* (Hb^−^Mb^−^) and *N. coriiceps* (Hb^+^Mb^+^) because too few *C. rastrospinosus* (Hb^−^Mb^+^) were caught. In addition, hearts from both *N. coriiceps* and *C. rastrospinosus* were similarly tested at 4°C after an exposure period of a minimum of 5 days at 4 ± 0.5°C in 700 l insulated recirculating seawater tanks (4–6 fish per tank); fish were initially held at 0 ± 1°C for 24 h before increasing the temperature by 1°C daily using 3-kW Elecro Titanium inline heaters (Aqualogic, San Diego, CA, USA). *C. aceratus*, which was intolerant of more than 1--2°C rise in temperature, proved difficult to acclimate to 4°C.

### Measurements and analysis

Perfusate outflow was measured with an in-line Transonic probe (4.0 mm diameter) and flowmeter (T206, Transonic Systems, Ithaca, NY, USA), gravimetrically calibrated at 1°C and 4°C to adjust for temperature effects on factory-calibrated flow readings. P_i_ and P_o_ were measured near the input and output cannulae, respectively, *via* fluid-filled tubes connected to pressure transducers (DP6100, Peter von Berg, Medizintechnik, Germany) and referenced to saline level in the bath. Recorded pressures were corrected to provide true values using individual calibrations of cannula resistance. Flow and pressure signals were amplified (4CHAMP amplifier, Somedic, AB, Hörby, Sweden) and processed on-line (custom LabView program; National Instruments Sweden AB, Kista, Sweden) to display real time raw values of P_i_, P_o_, V_S_, $\dot{Q}$ and *f*_H_ (determined from the pulsatile flow trace) throughout the experiment. *W*_C_, the product of [$\dot{Q}$ (ml min^−1^) × (P_o_ − P_i_) × 0.00167]) was displayed on-line and used to determine maximum performance when P_o_ was elevated. Data were analysed off-line for 5–10 stable, consecutive heartbeats for each incremental change in P_i_ and P_o_. *W*_C_ was corrected for ventricular mass (mW g^−1^ ventricle mass), and $\dot{Q}$ was corrected for body mass (ml min^−1^ kg^−1^ body mass).

### Statistical analyses

All data are presented as mean ± standard error (s.e.m.), analysed using SPSS v.23 (SPSS Inc., Chicago, IL, USA). Intraspecific comparisons (temperature × adrenaline), or interspecific comparisons for similar exposure and test temperatures (species × temperature), used a two-way ANOVA with Tukey’s Kramer *post-hoc* tests. A one-way Welch’s ANOVA was used to compare differences between 1°C-acclimated animals tested at 1°C and 4°C, and between 4°C-exposed animals tested at 4°C; the dose-response to adrenaline was tested by one-way repeated measures ANOVA with Sidák *post-hoc* tests at each test temperature. Relative ventricular mass (RVM) was transformed by Lngamma function to achieve normality for statistical comparisons. Statistical significance was assigned to *P* ≤ 0.05.

### Chemicals

The physiological saline used for perfusate (pH 8.1 at 1°C) contained 250 mM NaCl, 2.5 mM KCl, 0.9 mM MgSO_4_.6H_2_O, 2.5 mM CaCl_2_.2H_2_O, 5.6 mM glucose, 3.9 mM TES free acid and 6.1 mM TES free base. Tricaine methane sulfonate (MS-222) and adrenaline bitartrate were from Sigma-Aldrich (St. Louis, USA).

## Results

### Morphometry

At ambient (holding) temperature, RVM (Table 1) was 30% larger in *C. rastrospinosus* (Hb^−^Mb^+^) than for *C. aceratus* (Hb^−^Mb^−^) (*P* < 0.05). RVM of both *C. aceratus* (3-fold) and *C. rastrospinosus* (4-fold) were significantly (*P* < 0.05) larger than *N. coriiceps* (Hb^+^Mb^+^), which was more typical of temperate teleosts. RVM values for *C. rastrospinosus* and *N. coriiceps* did not change significantly after a period of exposure to 4°C.

**Table 1:** Morphometric details of fish used for the perfused heart experiments (mean ± s.e.m.)

	Acclim. temp (°C)	Test temp (°C)	*N*	Body mass (g)	Total length (cm)	Relative atrial mass (%BM)	Relative ventricular mass (%BM)
*C. aceratus*	1	1	8	1054±123^a^	52±2^a^	0.107±0.011^a^	0.304±0.011^a^
	1	4	7	676±62^b^	44±1^b^	0.077±0.005^a^	0.253±0.011^a^
*C. rastrospinosus*	1	1	6	478±57^b^	40±3^b^	0.148±0.013^b^	0.395±0.029^b^
	4	4	5	350±79^c^	36±2^c^	0.077±0.014^a^	0.364±0.030^a^
*N. coriiceps*	1	1	7	1093±87^a^	40±1^b^	0.027±0.002^c^	0.098±0.003^c^
	1	4	6	1102±142^a^	41±2^b^	0.024±0.002^c^	0.097±0.007^c^
	4	4	6	1217±102^a^	42±1^b^	0.025±0.004^c^	0.089±0.003^c^

Acclim, acclimation; temp, temperature; BM, body mass. A dissimilar letter indicates significant difference (*P*<0.05), tested by one-way ANOVA.

### Maximum cardiac performance tested at 1°C

#### *C. aceratus* (Hb^−^Mb^−^)

**Figure 1:**
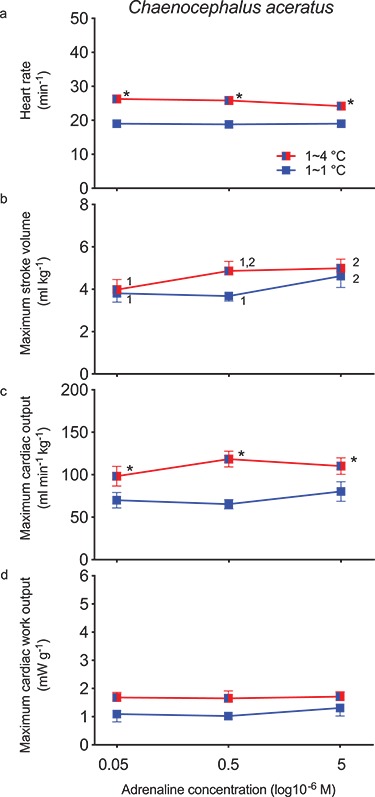
Maximum cardiac performance of the *in situ* perfused heart of *C. aceratus* (Hb^−^Mb^−^) acclimated to 1°C as a function of adrenaline concentration in the perfusate, tested at two temperatures. A significant difference between adrenaline concentrations is indicated by dissimilar numbers; an asterisk indicates a significant difference between the performance at 1°C (*n* = 8) and 4°C (*n* = 7) for a given adrenaline concentration.

Initial *f*_H_ (19.0 ± 0.8 min^−1^) was unresponsive to further adrenergic stimulation (Fig. 1), which improved some indices of maximum cardiac performance. For example, 5 μM adrenaline generated a maximum *V*_S_ of 4.6 ± 0.5 ml kg^−1^, a maximum $\dot{Q}$ of 80.2 ± 11.6 ml min^−1^ kg^−1^ and a maximum *W*_C_ of 1.3 ± 0.3 mW g^−1^. By comparison, maximum *V*_S_ (3.8 ± 0.4 ml kg^−1^) with 50 nM adrenaline was significantly (*P* < 0.05) lower, but maximum $\dot{Q}$ (70.0 ± 9.1 ml min^−1^ kg^−1^) and maximum *W*_C_ (1.1 ± 0.3 mW g^−1^) were unchanged.

#### *C. rastrospinosus* (Hb^−^Mb^+^)

**Figure 2:**
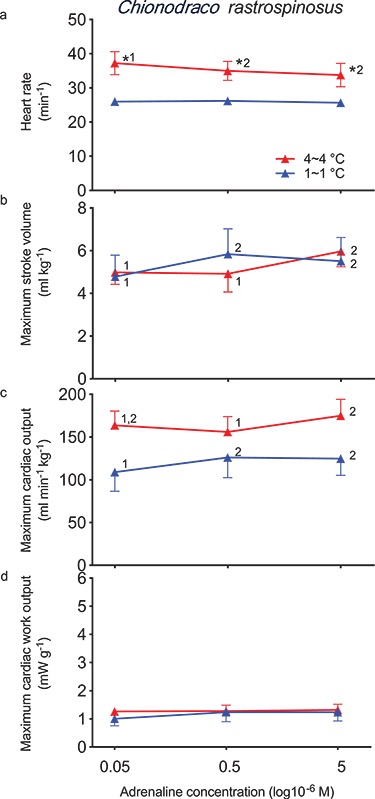
Maximum cardiac performance of the *in situ* perfused heart of *C. rastrospinosus* (Hb^−^Mb^+^) acclimated to 1°C and after an acclimation period at 4°C as a function of adrenaline concentration in the perfusate and tested at two temperatures (1°C and 4°C; *n* = 6 and 5, respectively). Significant differences are identified as Fig. 1.

Initial *f*_H_ (26.0 ± 0.5 min^−1^) was also unresponsive to further adrenergic stimulation (Fig. 2) that, as with *C. aceratus*, improved some indices of maximum cardiac performance. For example, 5 μM adrenaline generated a maximum *V*_S_ of 5.8 ± 1.1 ml kg^−1^, a maximum $\dot{Q}$ of 126 ± 23 ml min^−1^ kg^−1^ and a maximum *W*_C_ of 1.2 ± 0.3 mW g^−1^. Maximum *V*_S_ (4.8 ± 1.0 ml kg^−1^) and maximum $\dot{Q}$ (109 ± 22 ml min^−1^ kg^−1^ with 50 nM adrenaline was significantly (*P* < 0.05) lower, but maximum *W*_C_ (1.0 ± 0.3 mW g^−1^) was unchanged.

#### *N. coriiceps* (Hb^+^Mb^+^)

**Figure 3:**
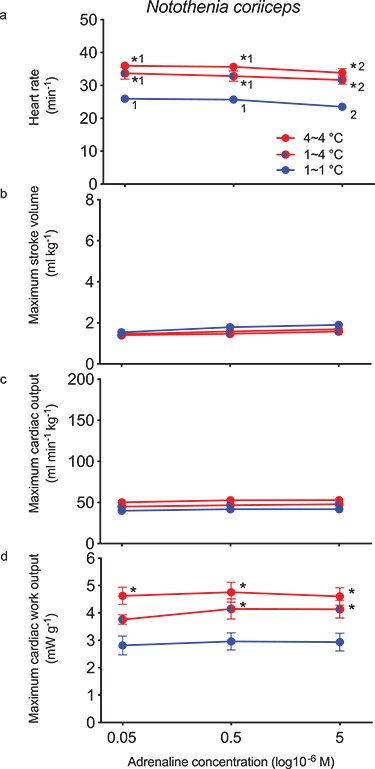
Maximum cardiac performance of the *in situ* perfused heart of *N. coriiceps* (Hb^+^Mb^+^) as a function of the adrenaline concentration in the perfusate. Fish were acclimated either to 1°C and tested at 1°C (*n* = 7) and 4°C (*n* = 6), or exposed to 4°C for an acclimation period and tested at 4°C (*n* = 6). Significant differences are identified as Fig. 1.

In contrast to icefishes, 5 μM adrenaline significantly decreased initial *f*_H_ (26.0 ± 0.9 to 23.5 ± 1.0 min^−1^; *P* < 0.05, Fig. 3), but maximum *V*_S_ (1.9 ± 0.1 ml kg^−1^), maximum $\dot{Q}$ (40.0 ± 2.3 ml min^−1^ kg^−1^) and maximum *W*_C_ (2.8 ± 0.3 mW g^−1^) were not significantly different compared with 50 nM adrenaline.

#### Interspecific comparisons of maximum performance with 5 μM adrenaline at 1°C

**Figure 4:**
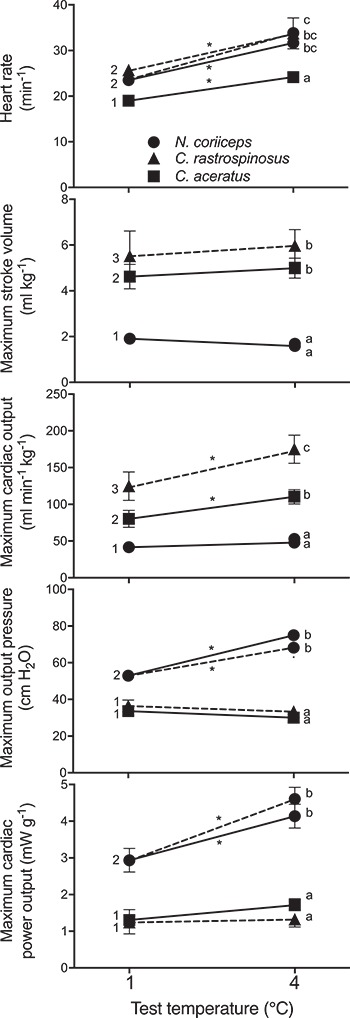
A comparison of the maximum cardiac performance of *in situ* perfused hearts of *C. aceratus* (Hb^−^Mb^−^; solid square), *C. rastrospinosus* (Hb^−^Mb^+^; solid triangle) and *N. coriiceps* (Hb^+^Mb^+^; solid circle) as a function of test temperature, using 5 μM adrenaline in the perfusate. Dashed lines connect fish tested at their acclimation temperature (either 1°C or 4°C), solid lines connect fish acclimated to 1°C and acutely warmed to 4°C for testing. A significant difference is indicated by dissimilar numbers between species when tested at 1°C, by dissimilar lower letters between species when tested at 4°C and by an asterisk between performance at 1°C and 4°C for each species.

*f*_H_ was significantly (*P* < 0.05) lower in *C. aceratus* (Hb^−^Mb^−^) than in either *C. rastrospinosus* (Hb^−^Mb^+^) or *N. coriiceps* (Hb^+^Mb^+^), whose *f*_H_ was not significantly different from each other (Fig. 4). Maximum *V*_S_ and maximum $\dot{Q}$ were highest for *C. rastrospinosus*, intermediate for *C. aceratus* and lowest for *N. coriiceps*. *N. coriiceps* generated a significantly (*P* < 0.05) and substantially higher maximum P_o_ and maximum *W*_C_ than both icefish species, which were similar to each other in this regard.

### Response of maximum cardiac performance on acute warming to 4°C

#### *C. aceratus* (Hb^−^Mb^−^)

Both *f*_H_ (26.3 ± 1.1 min^−1^) and maximum $\dot{Q}$ (98.3 ± 11.6 ml min^−1^ kg^−1^) measured at 4°C with 5 μM adrenaline were significantly (*P* < 0.05) higher when compared to 1°C (Fig. 1). Even so, maximum *V*_S_ (5.0 ± 0.4 ml kg^−1^) and maximum *W*_C_ (1.7 ± 0.2 mW g^−1^) did not change significantly. By comparison, maximum *V*_S_ was significantly (*P* < 0.05) lower (4.0 ± 0.5 ml kg^−1^) with 50 nM adrenaline, similar to the situation at 1°C, but the initial *f*_H_, maximum $\dot{Q}$ and maximum *W*_C_ were unchanged.

#### *N. coriiceps* (Hb^+^Mb^+^)

Both *f*_H_ (31.7 ± 1.4 min^−1^) and maximum *W*_C_ (4.1 ± 0.3 mW g^−1^), but not maximum *V*_S_ (1.6 ± 0.2 ml kg^−1^) or maximum $\dot{Q}$ (48 ± 4 ml min^−1^ kg^−1^), with 5 μM adrenaline were significantly (*P* < 0.05) higher with acute warming to 4°C when compared with 1°C (Fig. 3). The heart could also generate a significantly higher maximum P_o_ (76 cm H_2_O *vs.* 53 cm H_2_O; Fig. 4). Adrenergic stimulation had no significant effects on maximum *W*_C_, maximum *V*_S_ or maximum $\dot{Q}$ at 4°C, but initial *f*_H_ (33.8 ± 2.1 min^−1^) was significantly (*P* < 0.05) decreased by a small amount.

#### Interspecific comparisons with acute warming to 4°C

Similar to 1°C, *f*_H_ remained significantly (*P* < 0.05) lower in *C. aceratus* than in *N. coriiceps*, while maximum *V*_S_ and $\dot{Q}$ remained significantly (*P* < 0.05) higher for *C. aceratus* than *N. coriiceps* (Fig. 4). Again, *N. coriiceps* (Hb^+^Mb^+^) generated a significantly (*P* < 0.05) higher maximum P_o_ and maximum *W*_C_ than *C. aceratus* (Hb^−^Mb^−^).

### Maximum cardiac performance after 5 days exposure to 4°C

#### *C. rastrospinosus* (Hb^−^Mb^+^)

When 4°C-exposed fish were tested at 4°C, the initial *f*_H_ (37.3 ± 3.3 min^−1^) was significantly (*P* < 0.05) higher compared with fish acclimated to 1°C and tested at 1°C (26.0 ± 0.5 min^−1^; Fig. 2). While 5 μM adrenaline produced a small, significant (*P* < 0.05) decrease in *f*_H_ (33.8 ± 3.4 min^−1^) at 4°C when compared with 50 nM adrenaline, maximum *V*_S_ and maximum $\dot{Q}$ increased significantly (*P* < 0.05) by a small amount without significantly changing maximum *W*_C_. Even so, neither maximum *V*_S_ (6.0 ± 0.7 ml kg^−1^), maximum $\dot{Q}$ (175 ± 19 ml min^−1^ kg^−1^), nor maximum *W*_C_ (1.3 ± 0.2 mW g^−1^) were significantly (*P* > 0.05) different at 4°C when compared with fish tested at 1°C.

#### *N. coriiceps* (Hb^+^Mb^+^)

When tested at 4°C, the initial *f*_H_ (36.0 ± 1.0 min^−1^) was significantly (*P* < 0.05) higher when compared with fish tested at 1°C (Fig. 3). Stimulation with 5 μM adrenaline had no significant effects on maximum *W*_C_, maximum *V*_S_ or maximum $\dot{Q}$, but produced a small, significant (*P* < 0.05) decrease in *f*_H_ when compared with 50 nM adrenaline. While maximum *W*_C_ (4.6 ± 0.3 mW g^−1^) with 5 μM adrenaline was significantly (*P* < 0.05) higher after a period of exposure to 4°C compared with fish acclimated to 1°C, neither maximum *V*_S_ nor maximum $\dot{Q}$ were different (*P* > 0.05). Moreover, none of the maximum performance parameters were different (*P* > 0.05) when 4°C-exposed fish tested at 4°C were compared with fish acclimated to 1°C and acutely warmed to 4°C (Fig. 4).

#### Interspecific comparisons with a period of exposure to 4°C

While 4°C-exposed *N. coriiceps* had a significantly (*P* < 0.05) higher maximum *W*_C_ and maximum P_o_ than 4°C-exposed *C. rastrospinosus*, maximum $\dot{Q}$ and maximum *V*_S_ were significantly (*P* < 0.05) lower (Fig. 4)*.* In general, cardiac performance at 4°C was similar whether acutely exposed to this temperature change or measured after a period of exposure to 4°C.

## Discussion

### Maximum cardiac performance

We provide the first estimate of maximum cardiac pumping capacity for an icefish that lacks Mb, and the first comparison of maximum *in situ* cardiac performance among notothenioids differing in their expression of Hb and Mb. This comprehensive study of maximum cardiac function of notothenioid fishes now allows a better assessment of the likely scope for acclimation during Southern Ocean warming. While we show that *N. coriiceps* had a robust cardiac performance at all of the temperature conditions we tested, the extremely large hearts associated with loss of Hb expression in icefishes incur a cost to maximum *W*_C_, which was considerably lower than in *N. coriiceps*. While the low vascular resistance of icefish (e.g. in *C. hamatus* it is only 10–20% that of Hb^+^ fishes; Acierno *et al*., 1997) will offset this to an extent, low *W*_C_ may still limit scope for activity despite an unusually high maximum $\dot{Q}$ near 0°C. In this regard, the additional loss of Mb in *C. aceratus* may create a more precarious situation than *C. rastrospinosus* in a warmer world. Specifically, the maximum $\dot{Q}$ values recorded here are in the lower range of those previously published for icefishes (70–300 ml min^−1^ kg^−1^; Axelsson, 2005). Moreover, a functional consequence for the evolutionary loss of Mb may be our novel finding of a significantly higher maximum $\dot{Q}$ at 1°C for *C. rastrospinosus* (Hb^−^Mb^+^; 128 ml min^−1^ kg^−1^) than for *C. aceratus* (Hb^−^Mb^−^; 80 ml min^−1^ kg). Routine *Q in vivo* is 27 ml min^−1^ kg^−1^ and maximum $\dot{Q}$ 80 ml min^−1^ kg^−1^ for *C. aceratus* at 1°C (Joyce *et al*., 2018b), suggesting that cardiac factorial scope could be as high as 3 (i.e. 80/27) and similar to other teleosts (Farrell and Smith, 2017). However, routine *Q in vivo* for *N. coriiceps* at 1°C is 7 ml min^−1^ kg^−1^, while the maximum $\dot{Q}$ is 40 ml min^−1^ kg^−1^, suggesting a higher cardiac factorial scope of 5.7. These comparisons suggest that the red-blooded *N. coriiceps* has the potential to withstand a warming climate better than icefishes, where conservation efforts within the Southern Ocean should be focused.

### Species comparisons of maximum performance

*In vitro* measurements previously showed that $\dot{Q}$ in icefishes at −0.5°C (*V*_S_ ~ 3.5 ml kg^−1^) declined appreciably when afterload was increased to > 30 cm H_2_O (Acierno *et al*., 1997; *cf*. maximum P_o_ in the present study ~ 35 cm H_2_O). Interestingly, poisoning Mb with nitrite in *C. rastrospinosus* (Hb^−^Mb^+^) significantly reduced *W*_C_ to 0.8 mW g^−1^(P_o_ = 29 cm H_2_O) from 1.1 mW g^−1^ (P_o_ = 31 cm H_2_O), whereas *W*_C_ in *C. aceratus* (Hb^−^Mb^−^) was unaffected and remained at 1.4 mW g^−1^ with a similar afterload (Acierno *et al*., 1997). Unfortunately, this study did not assess maximum $\dot{Q}$, but the present findings suggest that *C. aceratus* may have been working near maximum, unlike *C. rastrospinosus*. Previous *in vitro* estimates of maximum $\dot{Q}$ in an icefish are limited to those for *C. hamatus* (Hb^−^Mb^+^) at 3°C (200–300 ml min^−1^ kg^−1^ and maximum *V*_S_ of 8–11.5 ml kg^−1^; Tota *et al*., 1991). Our *in situ* values for maximum $\dot{Q}$ at 4°C were considerably lower for *C. rastrospinosus* (Hb^−^Mb^+^; 175 ml min^−1^ kg^−1^) and still lower in *C. aceratus* (Hb^−^Mb^−^; 98 ml min^−1^ kg^−1^); whether this reflects an important species variation or methodological differences is unclear. However, an *in situ* heart preparation is likely a superior preparation to placing a heart in an artificial pericardium, as potential damage during isolation is avoided. As observed, icefish hearts are easily overstretched by excessive filling pressure even when the pericardium is intact, resulting in a spontaneous decrease in *f*_H_. In addition, *in vitro* heart perfusions place an input cannula in the atrium at the sino-atrial junction, potentially impairing activity of pacemaker cells located there (Haverinen and Vornanen, 2007; Farrell and Smith, 2017); intrinsic *f*_H_ for isolated heart preparations was far more variable for *C. aceratus* at −0.5°C (22–30 min^−1^; Acierno *et al*., 1997) and *C. hamatus* at 0.1°C (19.5–26.3 min^−1^; Tota *et al*., 1991) compared with the stable initial *f*_H_ of our study. Notably, neither Tota *et al*. (1991) nor Acierno *et al*. (1997) used adrenaline in their *in vitro* studies, although it is plausible that catecholamines released from cardiac tissues could have accumulated in recycled perfusate.

Both *C. hamatus* and *C. rastrospinosus* have a much higher maximum *V*_S_ compared with *C. aceratus*, a difference that may be related to Mb expression facilitating a somewhat higher cardiac afterload, while a 30% larger relative ventricular mass and a ~ 60% higher maximum $\dot{Q}$ at 1°C (*C. rastrospinosus* vs. *C. aceratus*; above) are also of potential benefit. Although both these species have similar values of maximum P_o_ and W_C_, Mb has a much higher oxygen affinity than Hb and enhances both oxygen storage and diffusion rate within cells. Other species differences among notothenioids also might affect maximal cardiac performance, e.g. icefish hearts lack mitochondrial creatine kinase (O'Brien *et al*., 2014) and energy charge of *N. coriiceps* hearts is typically higher than *C. aceratus* (O’Brien *et al*., 2018). In addition, the Mb^+^ state (independent of Hb loss) is associated with a 22% higher initial *f*_H_ at 1°C (26 min^−1^ in *C. rastrospinosus* vs. 19 min^−1^ in *C. aceratus*), a difference also seen at 4°C and between *N. coriiceps* and *C. aceratus*. Because the sino-atrial node was intact in the *in situ* preparation, we are confident that *f*_H_ was driven by a steady pacemaker activity, justifying our novel suggestion of a species difference in initial *f*_H_ among icefishes. A similar species difference was observed *in vivo* after blockade of cardiac vagal tone (24 min^−1^ for *N. coriiceps* and 14.2 min^−1^ for *C. aceratus*) (Joyce *et al*., 2018b), while initial *in situ f*_H_ for *C. aceratus* (19.0 min^−1^) compares well with *in vivo f*_H_ measured immediately following activity (17.6 min^−1^; Joyce *et al*., 2018b). Of potential value in setting resting metabolic rate, and hence scope for activity, comparisons of initial *f*_H_ values are confounded by the tonic level (50 nM) of adrenaline used to stabilize the *in situ* heart preparation; *f*_H_ of *C. aceratus* may simply be less sensitive to tonic adrenergic stimulation at concentrations < 50 nM than *C. rastrospinosus* (both species were unresponsive to further additions of adrenaline). In contrast, with a similar preparation, *M. scorpius f*_H_ increased by 22% at 1°C and by 27% at 6°C with maximal adrenergic stimulation, stimulatory effects that were fully reversed by β-adrenergic blockade (Farrell *et al*., 2013).

Maximum *W*_C_ of *N. coriiceps* at 1°C was impressive when compared with those of temperate-water teleosts at much warmer temperatures (Farrell and Jones, 1992; Axelsson, 2005; Farrell and Smith, 2017), maintaining maximum $\dot{Q}$ against a comparable afterload. Indeed, it shows a remarkably similar maximum cardiac performance to *M. scorpius* (Hb^+^Mb^+^) from Greenland, with comparable adaptive response observed on acute warming (Farrell *et al*., 2013). Despite a lower maximum *V*_S_ and $\dot{Q}$, *N. coriiceps* hearts generated more than twice the relative work output compared with both *C. rastrospinosus* and *C. aceratus*, in part due to an enhanced pressure-generating ability compared with icefish hearts, as reported earlier (Acierno *et al*., 1997). However, with the much larger ventricle of icefishes, absolute maximum cardiac work output for a 1 kg representative fish was similar for all three species. Whether this translates to a similar resilience to environmental change will depend on the origin of physiological challenges to be overcome.

Fish hearts are exquisitely sensitive to cardiac filling pressure (venous return), and perfused hearts can generate an output even with a sub-ambient P_i_ (*C. hamatus*: Tota *et al*., 1991; *C. aceratus*: Acierno *et al*., 1997 and present study; *C. rastrospinosus* and *N. coriiceps*: present study). However, neither icefish species examined here displayed the sensitivity to preload shown by *C. hamatus* (Tota *et al*., 1991), which increased *V*_S_ up to 11.5 ml kg^−1^ ($\dot{Q}$ up to 300 ml min^−1^ kg^−1^) with just a 0.25 cm H_2_O (≈0.025 kPa) increase in P_i_, although why this difference exists is unclear. Adrenergic stimulation can increase sensitivity to P_i_ (Farrell and Jones, 1992), as seen in the present study and with *M. scorpius* (Farrell *et al*., 2013), by increasing maximum *V*_S_. This effect may be particularly important for icefishes where high concentrations of nitric oxide resulting from lack of Hb conversion to nitrate (Beers *et al*., 2010) may reduce adrenergic-stimulated contractility *in vivo* (Rastaldo *et al*., 2007). The importance of adrenergic stimulation for cardiac contractility has been demonstrated in notothenioids: maximum tension of isolated ventricular strips from *C. aceratus* increased by 47% with adrenaline concentrations similar to those of the present study, while *N. coriiceps* maximum tension increased by a remarkable 377% with 1 mM adrenaline (Skov *et al*., 2009). These positive inotropic effects were more marked and species-specific than the modest stimulatory effects of adrenaline on *V*_S_ or $\dot{Q}$ (20–30% increases) seen *in situ*, so may reflect a potentially powerful role for tonic adrenergic stimulation of contractility not fully realized *in vivo*. Differences in adrenergic sensitivity could reflect species difference as well as thermal effects: adrenaline responsiveness increased at 4°C in *N. coriiceps* (present study) and at 6°C in *M. scorpius* (Farrell *et al*., 2013), although a dependency on tonic adrenergic stimulation existed in rainbow trout (*Oncorhynchus mykiss*) hearts at 5°C, but not at higher temperatures (Graham and Farrell, 1989). Ventricular strips of both *C. aceratus* and *N. coriiceps* developed alternans at high-pacing frequencies, an impairment exacerbated by adrenaline (Skov *et al*., 2009). A protective effect may be provided by the modest negative chronotropic effect of adrenaline, seen in both *N. coriiceps* and *C. rastrospinosus* at 4°C (present study), perhaps due to a stimulation of cardiac α-adrenoceptors or a prolongation of the ventricular action potential (Farrell and Jones, 1992). Clearly, the subtle differences in adrenergic stimulation of the heart that exist among polar fish species need further study to better understand their autonomic cardiac regulation, as sympathovagal balance helps define a species ecotype (Campbell *et al*., 2009).

### Temperature effects

The present study revealed a clear (30–40%) increase in *f*_H_ when notothenioid species were tested at 4°C, independent of whether the fish had been acutely warmed or experienced an exposure period at 4°C. Temperature has a variety of direct cardiovascular effects, but perhaps the most important and universal is an increase in *f*_H_ during acute warming (e.g. Sandblom and Axelsson, 2007; Farrell, 2009; Eliason *et al*., 2011; Keen and Gamperl, 2012). Notothenioids are clearly no exception in this regard, e.g. isolated *C. hamatus* hearts warmed from 0.6°C to 5.8°C, increased *f*_H_ by 21% to 24 min^−1^ (Tota *et al*., 1991) and *C. aceratus* doubled *f*_H_ to 36 min^−1^ when exposed for 48 h to 10°C (Hemmingsen *et al*., 1972). Routine *f*_H_ and $\dot{Q}$ both increased with acute warming from −1°C to 8°C in another Antarctic fish, *Pagothenia borchgrevinki* (Franklin *et al*., 2007). However, its scope to increase *f*_H_, *V*_S_ and $\dot{Q}$ following burst activity all decreased with increasing temperature because the increase in maximum cardiac capacity did not match the greater routine oxygen uptake. In the present study, both *C. aceratus* and *N. coriiceps* increased *f*_H_ by a similar amount when acutely warmed. Notably, only *N. coriiceps* significantly increased *W*_C_ (by 64%), largely through an increase in maximum P_o_, while *C. aceratus* maintained maximum *V*_S_ and maximum P_o_, but increased maximum $\dot{Q}$. Interestingly, the cardiac response to warming in *N. coriiceps* was similar to *M. scorpius*, which is considered a temperature generalist (Franklin *et al*., 2007) and maintained scope for $\dot{Q}$ with forced activity over a 10°C acute warming, whereas two closely related Arctic sculpins with a more restricted range could not do so (Franklin *et al*., 2013). These comparisons suggest that the ability to tolerate ocean warming may differ even among red-blooded polar species, as well as among icefishes.

The period of exposure used here was short relative to the slow responses known for some Antarctic fishes (Franklin *et al*., 2007; Franklin and Seebacher, 2009; Egginton and Campbell, 2016), even though they involved only small temperature changes. Therefore, conclusions concerning cardiac thermal acclimation for *C. rastrospinosus* and *N. coriiceps* must be treated with caution. For example, acclimation of *P. borchgrevinki* (Hb^+^Mb^+^) to 4°C for 4–5 weeks reduced peak $\dot{Q}$ after exercise at −1°C (factorial scope of 1.4 *vs*. 2.6 for −1°C acclimated fish), while peak $\dot{Q}$ was maintained on acute exposure to 4°C and 8°C (Franklin *et al*., 2007). Thermal compensation maintained cardiac performance at the new acclimation temperature and allowed scope for $\dot{Q}$ change up to 8°C, but at the expense of reduced cardiac performance when acutely cooled to −1°C (Franklin *et al*., 2007). For *N. coriiceps* maximum performance with acute warming and exposure to 4°C were not statistically different; in contrast, thermal tolerance increased by 1.2°C after just 7 days at 4°C (Bilyk and DeVries, 2011). Like acute warming of *C. aceratus*, *C. rastrospinosus* maintained maximum *V*_S_, maximum $\dot{Q}$ and maximum P_o_ after exposure at 4°C (present study). Clearly, a more extensive exploration of thermal acclimation effects in polar fishes is warranted, which may allow the capacity for different niche selection to be predicted, although facilities in remote areas remain a challenge for such detailed studies.

It is possible that over evolutionary time icefishes could accommodate warming as the Patagonian icefish *Champsocephalus esox* apparently has done, where surface water temperature is 10–12°C over its summer range. In addition, some notothenioids have escaped the stable cold waters of the Southern Ocean and successfully colonized warmer temperate water. Stranded following a northerly extension of the ice front the black cod, *Notothenia angustata*, has established a viable population around the southern coast of New Zealand.

## Concluding remarks

We provide robust estimates of maximal cardiac performance in two species of Channichthyid icefishes lacking facilitated systemic oxygen transport. While exhibiting the expected rise in heart rate on warming, our results indicate a potential to accommodate some degree of climatic change, which would have significant bearing on species survival in a warming world. While the loss of Hb is associated with large ventricles that deliver high cardiac output, retention of Mb allows additional improvements in cardiac function. Although both icefish species increased cardiac pumping capacity when warmed to 4°C, their hearts were unable to tolerate a high afterload, suggesting limited capacity to cope with stressful situations where sympathetic drive may increase peripheral resistance. However, during acute warming with activity *C. aceratus* increased *in vivo *$\dot{Q}$ 3-fold and vascular conductance 5-fold, suggesting that short-term cardiovascular compensation is possible (Joyce *et al*., 2018b). In contrast, a closely related species expressing both Hb and Mb was capable of higher maximum work capacity, an enhanced performance that may convey greater resilience to near-future ocean warming. Although these data demonstrate a limited capacity to withstand more frequently occurring short-term thermal extremes, and perhaps the potential to accommodate a gradual warming of the Southern Ocean, long-term temperature acclimation experiments are required to understand interspecific limits for stock conservation.

This novel information allows greater insight into plasticity of fish species previously considered to be living perilously close to their upper thermal limits as a consequence of unique physiological constraints. The issue is of some urgency, as they inhabit ocean regions around Antarctica that are experiencing the most dramatic effect of global warming; compelling reasons why we need to understand the ecophysiology of such species before conservation efforts become untenable. In addition, there has been relatively little research on thermal resilience of such important predators in the simplified food chain, and we provide the most reliable cross-specific comparison on maximal cardiac performance, a key factor in ecological fitness, to date.

## Supplementary material

Supplementary dataset supporting this article is available at Dryad: doi:10.5061/dryad.83vc5.
